# Reactions to Dream Content: Continuity and Non-continuity

**DOI:** 10.3389/fpsyg.2019.02676

**Published:** 2019-12-03

**Authors:** David Kahn

**Affiliations:** Department of Psychiatry, Harvard Medical School, Boston, MA, United States

**Keywords:** continuity, non-continuity, emotional reactions, dream-self, cognition

## Abstract

Although dream content may at times be quite outlandish or illogical, the dreamer’s emotional reactions to these events are not outlandish or illogical. Our study shows that the dreamer’s emotional reaction to people and events are similar to what they would have been in wake life. There is continuity between the emotional reactions of the dream and wake-self, even though situations may arise that are not likely or possible in wake life. For example, a dream may include people and places that span different times that are weaved together as if they were occurring at the moment. Further, the *behavior* of the dream-self is often different than that of the wake-self. When this happens, there is a non-continuity between the behavior of the dream and wake-self. Thus, there is both continuity and non-continuity between the dream and wake-self: Continuity in emotional reactions and non-continuity in the kinds of situations and behaviors that occur while dreaming. In the Kahn and Hobson, 2005a study, 58.7% of participants reported that their thinking *within* the context of the dream was similar to what it would have been had they been awake. About 55.1% of participants also reported that their thinking *about* the context of the dream was different than it would have been had they been awake. This difference affords the dream-self with novel experiences but that still elicit emotional reactions that are similar to how its wake-self would react. In essentially, every case when a comment was given to the question on thinking in the Kahn and Hobson, 2005a study, participants reported about how they emotionally reacted *within* the context of the dream and how they emotionally reacted *about* the content of the dream in comparison to how they would have reacted if awake.

## Introduction: Continuity and Non-Continuity

Cognition may be defined as the mental action or process of acquiring knowledge and understanding through thought, experience, and the senses. The question addressed in this article is how cognition is the same and how it changes when we are dreaming and how this change may be helpful to the dreamer when awake.

When we dream, there is both continuity and non-continuity between the dream-self and the wake-self. There is continuity of the dream and wake-self’s emotional reactions to events, people, and behavior. While implausible dream events often go unquestioned, the dreamer’s emotional reaction to these events within the context of the dream is similar to wake-life reactions. This similarity suggests that the core person, asleep or awake, reacts similarly.

Non-continuity is in the occurrence of unlikely or impossible dream events and behaviors where people and places that span different times and different ages may be weaved together as if they were occurring in the moment.

Non-continuity provides the dream-self with new situations, such as when the dream-self interacts with her 5-year-old son, who in wake life is 15 years old, or when the dream-self interacts with a deceased parent. Continuity is because one’s emotionaol reaction to the son or parent is similar to what they would have been in wake life even though the experience cannot occur when awake. Our wake and dream reactions remain tethered despite unlikely behaviors or logical inconsistencies in the dream.

The most common non-continuity between the dream and wake-self is in a character’s behavior, Kahn and Hobson (2003). An example of non-continuity of behavior between the dream and wake-self might be if the dream-self is having sex with someone he or she would not in wake life. Does the dream-self feel guilty? How does the dream-self react? How to not get caught? Even if the behavior of the dream-self is unlikely to happen in wake-life, the emotional reactions of the wake-self would be the same as that of the dream-self, feeling guilty, how to not get caught, etc.

Having both, continuity and non-continuity between our dream and wake lives, helps explain both the cognitive connection and the disconnection between our dream and wake worlds.

Continuity between our dream and wake worlds may also be said to occur if dreams simulate and prepare us for social engagement when awake as in the theory of Revonsuo et al. (2016) and Tuominen et al. (2019). Continuity can also help with emotional well-being, Pesant and Zadra (2006), Cartwright (2010), Walker and van der Helm (2009), Walker (2017). Another example of continuity between the wake and dream worlds is the retaining of a theory of mind while dreaming, i.e., knowing what our dream characters are thinking, Kahn and Hobson (2005b).

Previous studies that have examined different kinds of non-continuity include those of Horton (2017) who has argued that non-continuity in dreaming has a functional role in the process of memory consolidation by separating memories from their original contextual role. They are taken out of their original context and are newly integrated with existing memories “rendering salient aspects of those memories to become available for retrieval in isolation from their contextual features.”

Another benefit of non-continuity between the dream and wake-self is the making of unexpected associations and in the number and kind of associations that become linked during dreaming, e.g., see Horton and Malinowski (2015), Cai et al. (2009), Kahn and Gover (2010), Wagner et al. (2004), Stickgold et al. (1999). In the Stickgold et al. (1999) study, non-continuity between wake and dream sleep was explored through the use of a semantic priming task that measured the reaction time to find a word when it is preceded by a word that is associated with it. The study found that semantic priming was state dependent in that subjects awakened from REM sleep showed greater priming by weak primes than by strong ones during the REM carryover period. Subjects tested during normal wake hours showed greater priming by strong primes, as expected.

These unexpected and enhanced associations have been shown to enhance creativity in the wake state since creative problem solving improved if sleep contained REM, Cai et al. (2009). Subjects who had achieved REM sleep during a nap did better on a remote association task (RAT) than those who only had NREM or had no nap at all. These authors found that “REM enhances the integration of unassociated information for creative problem solving” (Cai et al., 2009).

Several other studies have demonstrated that after a night’s sleep there is an enhanced ability to find a hidden rule or obtain an insight that helps solve a difficult problem. After waking from REM sleep subjects were often able to solve problems that were intractable during the day, Wagner et al. (2004), Walker and Stickgold (2004), Walker et al. (2002).

Previous studies that have examined different kinds of continuity between dream and wake experiences include those by Schredl and Hofmann (2003), Hobson and Schredl (2011), Schredl (2000, 2003, 2006), Horton (2017), Horton and Malinowski (2011), Malinowski et al. (2014), Malinowski and Horton (2011, 2015), Kahn and Hobson (2003, 2005a), Kahan and LaBerge (2011), Kahan et al. (1997), Domhoff (2011), Kozmová and Wolman (2006), Samson and De Koninck (1986), Sutton et al. (1994), Llewellyn (2011, 2013).

### Dream Recall

Several of the above studies compared the ability to problem solve when awake with and without a previous nap or sleep containing REM. Several studies have also addressed the relevance of dream recall to enhanced problem solving after REM sleep, Blagrove (2007), Cartwright (2010), Schredl et al. (2003). Some of these have shown that dream recall, in fact, is relevant to problem solving. When we do recall dreams that have elements of a task previously learned, the dreams help improve performance on the previously learned task, Wamsley and Stickgold (2009, 2019). These authors showed that only when elements of the maze navigation task were incorporated in the dream did improvement occur after sleep.

Even if the dream is not recalled, the dream experience may still become a part of the general autobiographical and episodic memories that go into making up the core individual. The experience though not recalled into conscious awareness may become available on a non-conscious level as suggested in the study by Siclari et al. (2017). These authors found that high-frequency activity in prefrontal regions as occurs in dreaming “may mediate cognitive functions… such as encoding and storing long-term memories, … and planning and executing tasks.” Further, according to Hobson (2009), whether dream recall happens or not, sleep and dreaming provide a protoconsciousness that is fundamental toward the eventual development of full consciousness.

## Methodology

In this article, we have used the data from our original study on thinking (Kahn and Hobson, 2005a) to study the reactions of the dream-self to its own and to other dream characters’ actions. In the original study, we did not rely on outside judges to determine what the dreamer was or was not thinking. We asked the dreamer to say whether thinking had occurred and if so what its characteristics were. The advantages and disadvantages of this protocol have been previously discussed (Kahn et al., 2002).

For this study, we reexamined the submitted dream reports in the original 2005a study for instances when not only thinking but also emotional reactions to dream events and people were reported in their comments on thinking. In the original study about thinking, emotional reactions to events and people were not explicitly excluded. In fact, comments of participants almost always mentioned their emotional reactions along with their thinking in their comments.

### Sample Size

We first briefly review the sample and methodology of the original study. The sample consisted of 26 subjects, 24 young adults between the ages of 18 and 22 (15 women and 9 men attending a local college and not familiar with dream studies) and 2 older male adult subjects who are considered experts in dream studies.

The students submitted 151 dream reports over a 2-week period. The two older “dream expert” subjects submitted 27 dream reports over a 2-week period. The two population groups were combined since no substantial differences were found when the two population groups were considered individually.

The 26 participants submitted 178 dream reports in which there were 747 instances when thinking was reported to have occurred. Mean number of reports per subject was 6.8 (SD = 3.2). The mean number of words per report was 223 (SD = 112).

Upon awakening, participants in the study were asked to answer the question: would your thinking be the same as it was in the dream, if the event that occurred in the dream, occurred while awake? Please comment on how your thinking in the dream was the same or different than your thinking if awake.

Upon awakening, the participants were also asked to answer the question: “Would your thinking be the same regarding the occurrence of the event itself?”

## Results of the Original and Current Study

The answers to these questions from the 26 subjects were analyzed with the aid of the statistical package “Statview 5.0.” For each subject a mean value was calculated and from these an overall mean value. Most subjects replied that had their dream event occurred while awake, their thinking would have been the same as it was during the dream. The results are statistically significant. The majority of subjects also responded that their thinking *about* the occurrence of the event would not have been the same had they been awake. This difference was statistically significant to the *p* = 0.02 level. Thinking *within* the dream event was similar to waking but that thinking *about* the dream event was different. Subjects who said that their thinking *within* a dream event was the same as wake-state thinking, often went on to say that their thinking *about* the dream event was different than their wake state thinking. This combination was more than four times as common as its opposite; subjects rarely stated that their thinking within a dream event was different from their wake-state thinking and that their thinking about the dream event was the same as their wake-state thinking. All the above results were replicated when we calculated the two kinds of thinking on a per dream report basis as on the per dream event basis, reported above.

About 58.7% of the participants reported thinking *within* the context of the dream was the same, 32.9% reported thinking was different, and 6.4% could not decide. About 55.1% of the participants reported their thinking *about* dream context was different than it would have been had they been awake, 39.2% reported their thinking *about* dream context the same, and 3.5% could not decide.

**Figure 1:**
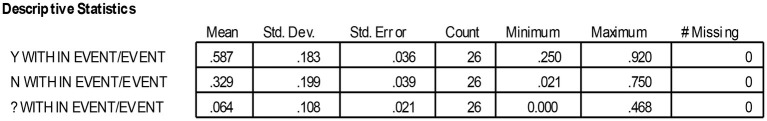
Thinking *within* a dream event. Descriptive statistics for the number of responses that thinking during a dream event would have been the same even if awake (Y within event/event), that thinking would have been different (N within event/event), and cannot decide if it would have been the same or not (? within event/event). The difference between subjects’ responses is highly significant based on a paired *t* test for significance between subjects’ responses from Kahn and Hobson (2005a).

**Figure 2:**
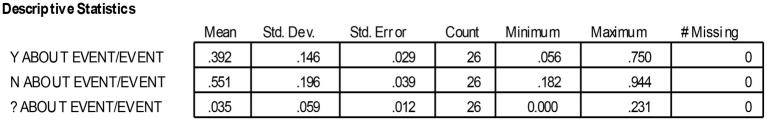
Thinking *about* a dream event. Descriptive statistics for the number of responses that thinking *about* the dream event would have been the same even if awake (Y about event/event), that thinking would have been different (N about event/event), and cannot decide if it would have been the same or not (? about event/event). The difference between subjects’ responses is significant based on a paired student *t* test for significance between subjects’ responses from Kahn and Hobson (2005a).

The results from the original Kahn and Hobson, 2005a study are summarized below in Figures 1 and 2, further details may be found in the original.

### Methodology in the Current Study

For the present study, we looked at the comments of the participants to these questions. It was discovered that almost all comments included participants’ emotional reactions not just their thinking.

Below are examples of participants’ answers to questions on thinking *within* and *about* a dream event when their emotional reactions were reported. These reactions both within and about a dream event are presented in tabular form (Table 1) below.

**Table 1:** Dream events, comments, and conclusions.

Participant	Dream event	Comment by participant	Conclusion
SF (m)	I was in my dorm room and I saw my roommate reclining on my bed. I saw this other guy, who started to molest him by running his fingers over his legs.	My roommate seemed to enjoy it, but I found it repulsive. “I was in a state of anger and that is how I would be in the same situation if I had been awake.”	The dream and wake-self emotionally react the same.
SF (m)	I was annoyed at the woman taking too long to make up her mind to climb the ladder to open the hatch to get into the restaurant.	If awake, I would have been equally annoyed at the woman being so slow to make up her mind. But, if awake SF goes on to say he’d recognize the improbability of climbing a ladder to enter the restaurant through a hatch door.	If awake, SF’s emotionl reaction would be the same as in the dream. Reaction *about* event is not the same.
JT (f)	We are on the way in and she is talking to me and says a swear word. I get really mad at her and call my dad to tell him what she just said, but he will not pick up his phone.	I would be mad at her if she swore when awake too. JT also said she would not call her dad if she did this.	Thus, JT’s disapproval of swearing did not change, but her calling her dad not likely to happen.
LG (f)	I am in a race with a fat girl. I am happy because I know I can beat her. I look back and see that it is a guy chasing me and I panicked. I ran inside a house to throw the guy off. I knew he did not know where I was and I thought I might win the race, especially when I saw he stopped to make clay sculptures.	“If awake I would have thought that the guy was weird for abruptly stopping to do clay sculptures in the middle of our race. In the dream I just accepted it as an opportunity to win the race.”	The dream self is happy to take advantage to win the race. Her desire to win is the same in waking and dreaming.
SF (m)	As I was retrieving the fallen paper towel, Dean R. was asking me about traveling and I replied by telling her about a trip I took to Washington DC	If, for whatever reason, Dean R. was talking to me, as awkward as I would feel, I would be as polite as I was in the dream.	Thus, the wake and dream-self emotionally act similarly. SF also said the event would be unlikely to happen.

In essentially every case, when a comment was given, participants reported about how they reacted emotionally. They reported how they emotionally reacted *within* the context of the dream and how they emotionally reacted *about* the content of the dream in comparison to how they would have reacted if awake. This approach should be validated in a future study that *directly* asks participants about their emotional reactions to events and people in the dream.

## Discussion

The Kahn and Hobson (2005a) study found that thinking by the dream-self *within* the context of the dream was similar to that of the wake-self. Thinking by the dream-self *about* dream content was different than that of the wake-self in that the dream-self did not question dream content as its wake-self would.

The current study found that emotional reactions of the dream-self were similar to how its wake-self would react. If the dream-self became impatient, angry, or happy about its own or a dream character’s behavior, its wake-self would have reacted the same. In general, participants reported that their emotional reactions *within* the context of the dream were similar to what its wake-self’s would have been. The study also found that reactions between the dream and wake-self were different *about* implausible dream content. Implausible content would have been noticed by its wake-self.

Thus, there is both continuity and non-continuity while dreaming. While the wake-self would not (or could not) engage in some of the behaviors of the dream-self, the result that emotional reactions of the dream-self and wake-self are similar shows that a continuity exists between the dream-self and the wake-self.

The non-continuity part is that the dream and wake-self’s reactions to unlikely and improbable events are different. However, bizarre upon awakening, *within* the dream, events go unquestioned and are accepted as occurring in the here and now. This acceptance is one reason that our emotional reaction to events and people within the dream are not different than when awake. The result that there is continuity between the dream-self and wake-self in their emotional reactions to events suggests that emotional reactions are state-independent, occurring in both the dream and wake states.

### Continuity, Non-continuity, Brain Changes, and Emergence

It is, in fact, remarkable that there is continuity between the dream and wake-self given how the brain’s chemistry and neural activity have changed from waking to dreaming and the fact that the emergent dream is different than its individual source elements, Kahn (2013, 2016). Serotonin and norepinephrine are absent in rapid eye movement (REM) dreaming, and reduced in NREM dreaming. No longer is the brain presented with a balanced concentration of cholinergic and aminergic chemistry, and no longer is the neural activity of the executive portions of the prefrontal areas as active as in the wake state, Braun et al. (1997), Maquet (2000), Cicogna and Bosinelli (2001), Hobson et al. (2000), Nir and Tononi (2010), Siclari et al. (2017). This study suggests that despite the brain changes that give rise to non-continuity between dream and wake content, continuity between the dream and wake-self’s emotional reactions *within* the context of the dream persists. This, in turn, suggests that core personality traits are state-independent.

## Data Availability Statement

The datasets generated for this study are available on request to the corresponding author.

## Author Contributions

The author confirms being the sole contributor of this work and has approved it for publication.

### Conflict of Interest

The author declares that the research was conducted in the absence of any commercial or financial relationships that could be construed as a potential conflict of interest.
